# The significance of diagnosing associated clonal mast cell diseases in patients with venom-induced anaphylaxis and the role of bone marrow investigation

**DOI:** 10.1186/2045-7022-3-22

**Published:** 2013-07-04

**Authors:** Theo Gülen, Barbro Dahlén, Birgitta Sander, Hans Hägglund, Gunnar Nilsson

**Affiliations:** 1 Department of Respiratory Medicine and Allergy, M53, Karolinska University Hospital Huddinge, SE-141 86 Stockholm, Sweden; 2 Department of Medicine Solna, Clinical Immunology and Allergy Research Unit, Karolinska Institutet, Stockholm, Sweden; 3 Department of Pathology, Karolinska University Hospital Huddinge, Stockholm, Sweden; 4 Department of Hematology, Karolinska University Hospital Huddinge, Stockholm, Sweden; 5 Mastocytosis Center Karolinska, Karolinska University Hospital and Karolinska Institutet, Stockholm, Sweden; 6 Centre for Allergy Research, Karolinska Institutet, Stockholm, Sweden

**Keywords:** Anaphylaxis, Hymenoptera sting, Mastocytosis, Mast cell clonality, Serum tryptase

## Abstract

Hymenoptera venom allergy (HVA) represents a particular risk for exceptionally severe anaphylactic sting reactions in patients with clonal mast cell disorders (CMD). Nevertheless, conventional investigations are not sufficient to do accurate risk assessments. Increased levels of baseline serum tryptase (sBT) (>11.4 μg/L) is highly associated with severe anaphylactic reactions and with a possible underlying CMD. The measurement of baseline serum tryptase, thus, has opened the possibility to screen for CMD. In the present study, we sought to investigate whether bone marrow evaluation provides more accurate diagnosis in patients with HVA.

Three patients of the same sex and similar age with HVA were enrolled in this clinical study. The patients underwent comprehensive allergy work-up including skin prick testing, measurements of serum total IgE concentrations and baseline serum tryptase. Bone-marrow biopsies were also performed in all three patients to assess underlying CMD.

We evaluated characteristics of the bone marrow mast cells by pathology, flow cytometry and detection of D816V mutation by using current WHO-criteria, which led to changes in the final diagnosis compared to the assessments done by classical allergy work-up and measurements of sBT. Three distinct diagnostic outcomes including systemic mastocytosis, monoclonal mast cell activation syndrome and non-clonal HVA were revealed.

We conclude that a bone marrow investigation is required for the correct diagnosis of hymenoptera venom-induced anaphylactic reactions in patients with elevated baseline tryptase levels (>11.4 μg/L), and this has important implications for management strategies.

## 

Hypersensitivity reactions after hymenoptera stings can be severe and potentially fatal. The special association between hymenoptera venom allergy (HVA) and mastocytosis has been long observed [1,2]. This concept has been broadened during recent years to encompass the larger disease entity of clonal mast cell disorders (CMD) [3], since a particular risk for severe, even fatal, anaphylactic sting reactions in patients with CMD have been reported [4,5].

The term CMD comprises systemic mastocytosis (SM) and monoclonal mast cell activation syndrome (MMAS) [6-8]. The common finding in these two conditions is the mast cells clonality; which can be proven by the detection of a codon 816 *KIT* mutation and/or occurrence of immunophenotypically aberrant mast cells expressing CD2 and/or CD25 [7,8]. In patients with MMAS, the WHO criteria for systemic mastocytosis are not met. Nevertheless, by using methods with higher sensitivity to detect bone marrow mast cells at low frequencies, and the *KIT* mutation analysis of purified mast cells could further improve the diagnosis [9].

Presently, the diagnosis of HVA is based on clinical history, skin prick test and allergen-specific IgE [10]. Moreover, the measurement of baseline serum tryptase (sBT) has opened the possibility to screen for CMD. However, conventional investigations, including allergy work-up and measurements of sBT levels, are not sufficient enough to predict a possible underlying CMD. In the present study, therefore, we sought to assess whether investigation with bone marrow biopsy and flow cytometry provides more accurate diagnosis in HVA patients with elevated baseline tryptase levels (>11.4 μg/L).

## Findings

### Patients and methods

Case 1: A 72 year old, previously healthy woman without known sensitization, had a wasp sting on her hand in 2001. She reacted only with local swelling. She got another wasp sting on her lower arm in 2003, and immediately noted swelling of the face. She felt tingling in hands and fingers, laid down on the ground and began to sweat. She had no breathing difficulties. She was about to pass out when the ambulance arrived. Her blood pressure was too low to measure. The patient received standard treatment with epinephrine and intravenous fluids by ambulance personal before she was transferred to hospital, where she remained overnight and recovered.

Case 2: A 67 year old, previously healthy woman without known sensitization, got a wasp sting in her lower leg during the 1970s and reacted only with local swelling. In 2005 she had another wasp sting on her left elbow. Within 5 minutes, she became dizzy and experienced palpitations and chills, and had abdominal cramps, nausea and vomiting. Shortly thereafter she became unconscious and suffered urinary and fecal incontinence. She was transferred to hospital by ambulance, where she received standard treatment with epinephrine, antihistamines and corticosteroids and recovered.

Case 3: A 71 year old, previously healthy woman had her first wasp sting at the age of 5. She passed out and was taken into hospital. During the 1970s, she received another wasp sting and again reacted with syncope. She was transferred to the local hospital and received treatment with epinephrine, antihistamines and corticosteroids before she recovered. More recently, in 2008, she became dizzy and had heart palpitations after yet another wasp sting. She also had breathing difficulties. She was afraid of treating herself with epinephrine autoinjector because of concern about its side effects, but after a couple of hours she recovered spontaneously.

All patients went through a complete clinical and physical examination along with an allergic work-up including skin prick testing (SPT) with commercial extracts (ALK-Abelló A/S, Horsholm, Denmark) of hymenoptera venom (honey bee and vespula). SPT was considered positive if the difference between the mean of the wheal's length and width and the negative control was at least 3 mm. The specific IgE antibody test (Immuno CAP Phadiatop®, ThermoFisher, Uppsala, Sweden) was also performed and considered positive for values ≥0.35 kU/L. Moreover, serum concentrations (μg/L) of baseline tryptase levels (ThermoFisher) and serum total IgE (kU/L) levels were determined by ImmunoCap Total IgE (ThermoFisher, Uppsala, Sweden) in all three patients according to manufacturer’s instructions.

In the third case, we have further investigated by component-resolved diagnosis of serum sIgE antibodies with purified and recombinant species-specific allergens of hymenoptera venom r Ves v1, r Ves v5 and r Api m1 IgE (ThermoFisher, Uppsala, Sweden). The presence of hymenoptera allergen through basophil allergen threshold sensitivity, CD-sens, was also performed by using commercial honey bee and vespula extracts (ALK-Abelló A/S, Horsholm, Denmark), as previously described [11]. Finally, intracutaneous tests (ICT) were performed using bee and vespid venom extracts (ALK-Abelló A/S, Horsholm, Denmark). A volume of 0.02 ml of allergen concentrations ranging from 10^-5^ - 10° μg/ml, as well as negative and positive control (histamine 0.1mg/ml) were administered simultaneously in the skin. A wheal of 5 mm in diameter with erythema after 15 min was regarded as a positive reaction [12].

Diagnosis of anaphylaxis was confirmed in all patients according to NIH criteria [13], when either reduced blood pressure or associated-symptoms like syncope/pre-syncope; and/or respiratory compromise or a laryngeal oedema were present accompanied by the involvement of the skin-mucosal tissue or gastrointestinal symptoms.

Mast cells in bone marrow biopsy sections were analyzed to evaluate underlying CMD following established criteria for morphology, histology and immunohistochemistry, flow cytometry, and mutational analysis [14-16]. Diagnosis of systemic mastocytosis was evaluated using current WHO criteria [6] (Table 1). Notably, none of the patients presented with urticaria pigmentosa (UP) like skin lesions.

**Table 1:** WHO diagnostic criteria for systemic mastocytosis*

	
Major criterion	
1.	Presence of multifocal, dense mast cell infiltrates (>15 in aggregates) in bone marrow or other extracutaneous organs
Minor criteria	
1.	>25% mast cells spindle-shaped or with abnormal morphology in bone marrow or other extracutaneous organs.
2.	Detection of *c-kit* point mutation at codon 816 in bone marrow, blood or extracutaneous organs
3.	Mast cells that coexpress CD117 with CD2 and/or CD25, in bone marrow or extracutaneous organs
4.	Persistent increased serum total tryptase level (>20 ng/ml)

*The diagnosis of systemic mastocytosis (SM) can be established if the major and one minor criterion or three minor criteria are fulfilled (adapted from reference 6). If the patients fulfill only one or two minor criteria (particularly criteria 2 or 3), which not sufficient for SM diagnosis, will be diagnosed as monoclonal mast cell activation syndrome.

The study was approved by Stockholm's Ethical Review Board, (Dnr: 2011/1750-31/3), and patients provided their informed consent.

## Results

Both SPT and specific IgE antibody test by ImmunoCAP were positive in Case 1 and 2. Nevertheless, in Case 3, SPT, ImmunoCAP, component-resolved analysis of hymenoptera venom r Ves v1, r Ves v5 and r Api m1 IgE (<0.10 kE/L), and also basophil activation test CD-sens for hymenoptera venom were all negative (<0.10 kE/L). However, further investigation with ICT gave a positive reaction for wasp venom at concentration of 10^-4^ μg/ml.

**Figure 1:**
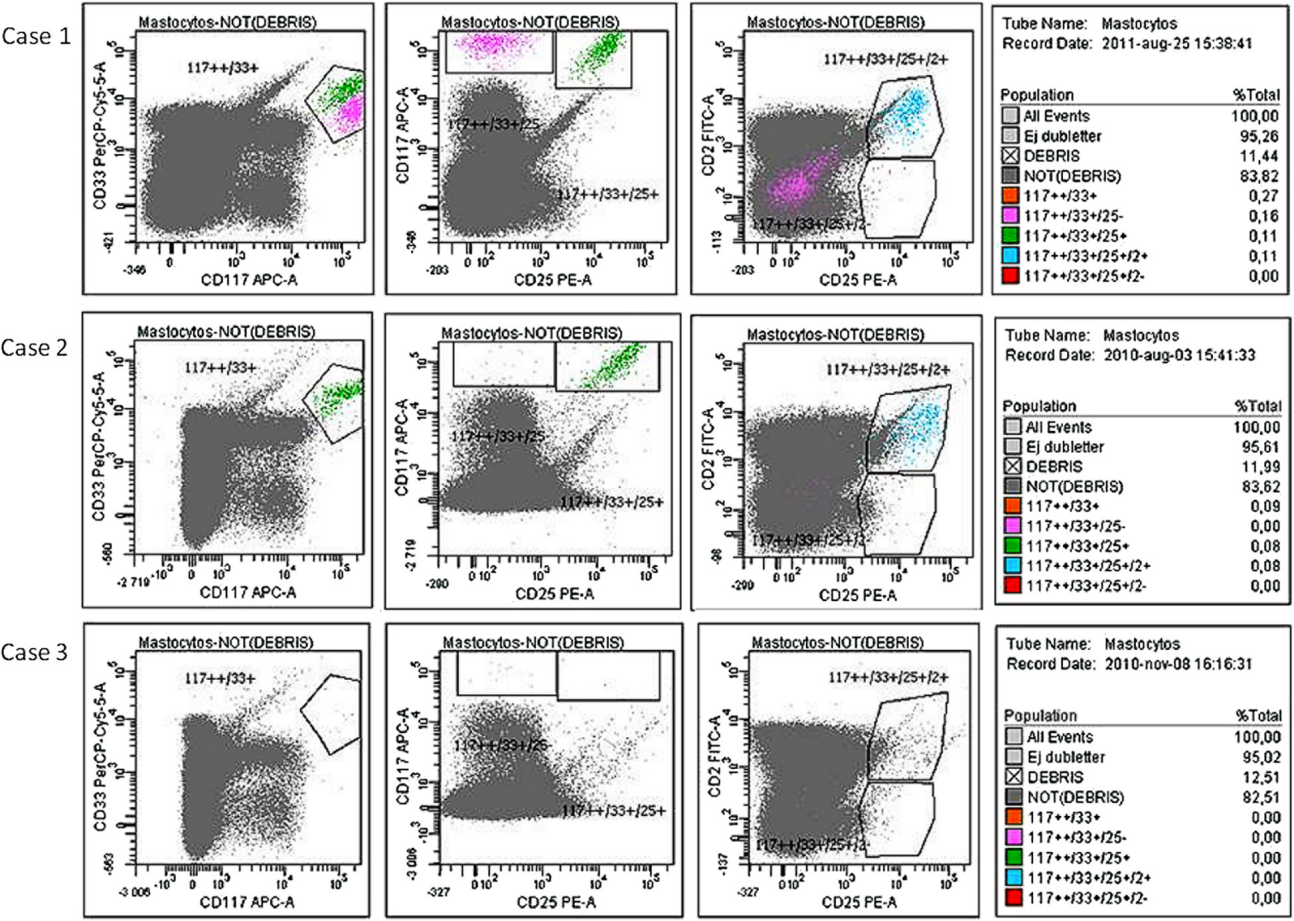
**Flow cytometric analysis of bone marrow aspirates in three cases.** The mast cell frequency and phenotype as evaluated by multiparameter flow cytometry. The percentages of CD117++/CD33+ mast cells and of mast cells with aberrant phenotype CD117++/CD33+/CD25+/CD2+ in the three cases are indicated in the graph. In case 1, 0.27% of total bone marrow cells were mast cells. These could be divided, based on expression of CD25 and CD2 in two populations; normal mast cells (pink) (0.16%) and pathological mast cells with expression of CD25 (green) and CD2 (blue) (0.11%). In case 2, total mast cells were 0.09% and the majority of these were CD25+/CD2+ (0.08%). In case 3, the frequency of mast cells was below the detection limit. The antibody combination used was CD2 (FITC; DAKO) - CD25 (PE; DAKO) - CD33 (PerCP-Cy5.5; Becton-Dickinson) - CD34 (PC7; Beckman-Coulter) - CD117 (APC; Beckman-Coulter) – HLA-DR (Pacific Blue; BioLegend) – CD45 (Krome Orange; Beckman-Coulter). 500 000 cells were collected using a Canto2 flow cytometer (BD Bioscience).

Histopathological, immunohistochemical and flow cytometry investigation of bone marrow led to changes in the final diagnosis compared to the assessments done by classical allergy work-up and measurements of serum baseline tryptase (sBT), and three distinct diagnostic outcomes were associated with the HVA. The MMAS diagnosis was made in Case 1 since only 2 minor criteria was fulfilled (aberrant phenotype and KIT D816V mutation); whereas Case 2 received diagnosis of SM by fulfillment of 3 minor criteria (atypical mast cell morphology, aberrant phenotype and Kit D816V mutation). However, there were no signs of mast cell clonality in Case 3. Figure 1 and Table 2 summarizes the different features of the patients.

**Table 2:** Summary of the investigations and demographic and clinical characteristics of patients

**Case No**	**1**	**2**	**3**
**Gender**	**Female**	**Female**	**Female**
**Current age (age with latest reaction)**	72 (63)	67 (60)	71 (67)
**Cause of anaphylaxis**	Wasp	Wasp	Wasp
**Symptoms during manifestation(s) of anaphylaxis**	Syncope, hypotension	Syncope, hypotension	Syncope, hypotension, dyspnea
**Skin prick test**	Vespula: Pos (2+)	Vespula: Pos (3+)	Vespula: Neg
	Honey bee: Neg	Honey bee: Neg	Honey bee: Neg
**ImmunoCap (normal value <0.35 kU/L)**	Vespula: 0.86 kE/L	Vespula: 0.76 kE/L	Vespula: <0.10 kE/L
	Honey bee: <0.10 kE/L	Honey bee: <0.10 kE/L	Honey bee: <0.10 kE/L
**Intracutaneous test**	n/a	n/a	Vespula: Pos (at 10^-4^ μg/ml concentration) Honey bee: Neg
**Serum total IgE (kE/L)**	14	89	35
**sBT (normal value <11.4 μg/L)**	12	14	23
**UP-like skin lesions**	None	None	None
**Multifocal mast cell clusters**	None	None	None
**>25% mast cells spindle- shaped or with abnormal morphology**	None	>25% spindle-shaped	None
**Abberant immunophenotype (CD2/CD25 mast cells)**	+/+	+/+	−/−
**Kit mutation D816V**	+	+	-
**Venom immunotherapy**	Yes, lifelong	Yes, lifelong	Planned for 5 years
**Final diagnosis**	MMAS^*^	SM^#^	Non-clonal HVA^¤^

*SM* systemic mastocytosis, *MMAS* monoclonal mast cell activation syndrome, *UP-like* urticaria pigmentosa like, *HVA* hymenoptera venom allergy.

^*^MMAS diagnosis was established by fulfillment of mast cells clonality (abberant immunophenotype and/or proven c-kit mutation D816V.

^#^SM diagnosis was established by fulfillment of 3 minor criteria (presence of atypical mast cell morphology, abberant immunophenotype and Kit mutation D816V).

^¤^Non-clonal HVA diagnosis was established after excluding mast cells clonality and other histopathological features of bone marrow mast cells.

n/a, not analyzed; *sBT* Baseline serum tryptase.

## Discussion

Evaluation of the above-mentioned cases exemplifies the high stakes diagnostic challenge encountered by the allergist. The patients presented with demographically (age and gender), clinically and somewhat serologically similar data, yet all three ended up with different diagnoses. Diagnosis of anaphylaxis could be made clinically by using NIH criteria, accordingly hypotension related symptoms presented in all cases. We have considered differential diagnosis such as hyperventilation, panic attack/anxiety; however, these were unlikely in the clinical context since there were a clear temporality in all instances. Unfortunately, there were no tryptase analyses from any of the cases during the acute reaction. There were no alternative explanations of elevated baseline tryptase levels in any of the patients since none of them suffered from associated diseases, such as renal failure.

It is noteworthy that the absence of venom sensitization by skin prick testing and/or ImmunoCap despite a convincing history has been a well-known phenomenon in mastocytosis patients with HVA [17,18]. In line with a recent study [19], even performing a basophil activation test (BAT) in the Case 3 did not provide any further support for wasp or bee sensitization. Interestingly, when we performed ICT in this patient, we could finally prove a wasp sensitization. Thus, all four tests (SPT, ImmunoCAP, BAT and ICT) should be performed in negative tested HVA patients before considering a patient as a true negative.

Patients with increased sBT (>11.4 μg/L) have a greater risk to develop more severe anaphylactic reactions to hymenoptera stings compared to those with normal values [20,21], and are also thought to have an increased risk for the appearance of side-effects during venom immunotherapy [22]. In Cases 1 and 2, fortunately both updosing and maintenance venom immunotherapies have been tolerated without any side effects (follow-up of seven years and three years respectively). Furthermore, the management of HVA patients with CMD involves unusual problems including severe, potentially fatal anaphylactic reactions after new stings, even if patients undergo venom specific immunotherapy [4,5]. The current guidelines, therefore, recommend life-long immunotherapy for patients with CMD, whereas 3–5 years venom immunotherapy induces long-term protection in patients with HVA alone. Thus, it is of immense importance to identify risk-prone patients by investigating a possible underlying CMD in patients with severe HVA.

Measurement of sBT provides a possibility to screen for patients with CMD; however, as demonstrated in this study, sBT *per se* is not a sufficient marker to distinguish different anaphylaxis phenotypes. Currently, the definite diagnosis in such patients can only be established after a bone marrow biopsy. Nevertheless, the identification of high risk patients that could be eligible for bone marrow examination remains a subject of discussion. According to the current guidelines, a bone marrow biopsy in patients with HVA is recommended if sBT is higher than 20 μg/L, or when signs and symptoms of mastocytosis, such as urticaria pigmentosa, are present [6]. Yet, 34% of the patients with HVA and CMD have a sBT lower than 20 μg/L [3]. Therefore it has been proposed that the sBT cut-off should be lowered to 11.4 μg/L to assess mast cell clonality including histopathological evaluation of bone marrow [22]. However, this practice has not yet come under consideration as a standard routine. Interestingly, the Spanish Network on Mastocytosis has recently proposed a scoring method to prescreen HVA patients before a possible further investigation with bone marrow examination in order to determine underlying CMD [23]. All three cases in our study scored enough to qualify bone marrow examination supporting that REMA-score could be beneficial tool in similar cases. In Case 3, we considered the option to perform a new bone marrow biopsy since the baseline tryptase levels remain elevated. At the moment, however, the patient denied to undergo a new biopsy.

The accurate diagnosis has both therapeutic and preventive implications for management strategies. Hence, patients with anaphylactic shock after insect sting and elevated sBT (>11.4 μg/L) might be considered for bone-marrow investigation in order to obtain better risk assessment regardless of other signs or symptoms of CMD.

## Abbreviations

HVA: Hymenoptera venom allergy; CMD: Clonal mast cell disorders; SM: Systemic mastocytosis; MMAS: Monoclonal mast cell activation syndrome; sBT: Serum baseline tryptase; SPT: Skin prick test; IgE: Immunoglobuline E; UP: Urticaria pigmentosa; BAT: Basophil activation test; ICT: Intracutaneous test.

## Competing interests

The authors report no conflict of interest.

## Authors’ contributions

TG wrote the manuscript. GN revised and critically reviewed the manuscript. TG, HH and BD performed clinical assessments of the patients included. BS carried out the evaluation of bone marrow samples. TG, BD, BS, HH and GN all contributed to the initiation and design of the study. All authors were involved in the preparation of the article and gave their final approval of the submitted version.
